# Antimetastatic Effects of Curcumin in Oral and Gastrointestinal Cancers

**DOI:** 10.3389/fphar.2021.668567

**Published:** 2021-08-11

**Authors:** Amirhossein Davoodvandi, Marjan Farshadi, Noushid Zare, Seyed Amirreza Akhlagh, Esmail Alipour Nosrani, Maryam Mahjoubin-Tehran, Parisa Kangari, Seyedeh Maryam Sharafi, Haroon Khan, Michael Aschner, Ghazaleh Baniebrahimi, Hamed Mirzaei

**Affiliations:** ^1^ Student Research Committee, Kashan University of Medical Sciences, Kashan, Iran; ^2^ Cancer Immunology Project (CIP), Universal Scientific Education and Research Network (USERN), Tehran, Iran; ^3^ Giamed Corp., Richmond Hill, ON, Canada; ^4^ Faculty of Pharmacy, International Campus, Tehran University of Medical Science, Tehran, Iran; ^5^ School of Medicine, Shiraz University of Medical Sciences, Shiraz, Iran; ^6^ Department of Nutrition, Science and Research Branch, Islamic Azad University, Tehran, Iran; ^7^ Department of Medical Biotechnology, Faculty of Medicine, Mashhad University of Medical Sciences, Mashhad, Iran; ^8^ Department of Tissue Engineering and Applied Cell Sciences, School of Advanced Medical Sciences and Technologies, Shiraz University of Medical Sciences, Shiraz, Iran; ^9^ Environment Research Center, Research Institute for Primordial Prevention of Non-Communicable Disease, Isfahan University of Medical Sciences, Isfahan, Iran; ^10^ Department of Pharmacy, Abdul Wali Khan University, Mardan, Pakistan; ^11^ Department of Molecular Pharmacology, Albert Einstein College of Medicine, Bronx, NY, United States; ^12^ Department of Pediatric Dentistry, School of Dentistry, Tehran University of Medical Sciences, Tehran, Iran; ^13^ Research Center for Biochemistry and Nutrition in Metabolic Diseases, Institute for Basic Sciences, Kashan University of Medical Sciences, Kashan, Iran

**Keywords:** gastrointestinal cancer (GI cancer), curcumin, therapy, metastasis, gastric cancer

## Abstract

Gastrointestinal (GI) cancers are known as frequently occurred solid malignant tumors that can cause the high rate mortality in the world. Metastasis is a significant destructive feature of tumoral cells, which directly correlates with decreased prognosis and survival. Curcumin, which is found in turmeric, has been identified as a potent therapeutic natural bioactive compound (Curcuma longa). It has been traditionally applied for centuries to treat different diseases, and it has shown efficacy for its anticancer properties. Numerous studies have revealed that curcumin inhibits migration and metastasis of GI cancer cells by modulating various genes and proteins, i.e., growth factors, inflammatory cytokines and their receptors, different types of enzymes, caspases, cell adhesion molecules, and cell cycle proteins. Herein, we summarized the antimetastatic effects of curcumin in GI cancers, including pancreatic cancer, gastric cancer, colorectal cancer, oral cancer, and esophageal cancer.

## Introduction

Various mechanisms are involved in the induction of malignant neoplasms in the gastrointestinal tract (Sharma and Bhatia, 2018). Most of the upper gastrointestinal tract carcinomas are caused by aggregated genetic events and uninterrupted mucosal injury (Sharma and Bhatia, 2018). While pre-existing adenomas are the origin of colorectal carcinomas, typically. Chronic injuries resulted in prolonged-mucosal damages such as inflammatory bowel disease (IBD). It causes a small number of cancers in the lower parts of the gastrointestinal tract (Axelrad et al., 2016). Research on the molecular pathways has led to considerable advances in the understanding of tumor progression processes. For example, detecting different mutations that affect tumor suppressor genes or those involved in DNA repair may be applicable for identifying the patients with heritable cancer risk (Wang et al., 2018). Besides, others show therapeutic indications. A growing number of oncogenic mutations have been recognized in gastrointestinal malignancies, which may be applied or manipulated (Lv, 2017).

Metastasis is disseminating tumoral cells from the original tumor to the primary organ's potential locations or other contiguous and remote organs (Das et al., 2020). As the significant destructive feature of tumoral cells, the incidence of metastasis in various types of cancer directly correlates with decreased prognosis and survival in patients (Deepak et al., 2020). Malignant cells dissemination of primary tumor is the first step for metastasis initiation, intravasation into the blood circulation system is considered the next step, the third step is linked to arrest of tumoral cells in a distant vascular bed, and in the fourth step, cancer cells extravasation into the interstitial tissue of a target organ occurs (Fouani et al., 2017). During the metastasis process and proliferation in a metastatic site, revascularization plays a crucial role in malignant spread, as it supplies the metabolic requirements for the fast duplicating malignant cells (Bielenberg and Zetter, 2015; Luo et al., 2020)**.**

Curcumin or [(1E,6E)-1,7-bis(4-hydroxy-3-methoxyphenyl)-1,6-heptadiene-3,5-dione] is a polyphenolic extraction of Curcuma longa species, which is often termed as turmeric (Shafabakhsh et al., 2019; Ashrafizadeh et al., 2020). Curcumin had been used as a traditional Ayurvedic medicine due to its significant anti-inflammatory (Satoskar et al., 1986), antioxidant (Masuda et al., 2001), and antimicrobial (Negi et al., 1999) properties. Currently, curcumin is associated with powerful anticancer properties. Different animal studies have shown that curcumin has important roles in inhibiting primary tumorigenesis in numerous organs as metastatic sites, such as mammary glands (Inano et al., 1999) and gastrointestinal tract (Huang et al., 1994). Some investigations showed that curcumin has potential regulatory effects on the expression of proangiogenic growth factors (6–8). Curcumin inhibits angiogenic activities caused by fibroblast growth factor (bFGF) in rabbit and mouse models (Mohan et al., 2000). It also diminished the vascular endothelial growth factor (VEGF) serum levels in mice models of hepatocellular carcinoma (Yoysungnoen et al., 2006). Interleukin (IL)-1β and monocyte chemotactic protein-1 (MCP-1) are critical inflammatory cytokines in tumorigenesis. Accordingly, some studies demonstrated that these cytokines' expression levels reduced after curcumin intervention (Abe et al., 1999).

The dynamic interplay between neoplastic cells and the immune microenvironment regulates multiple steps in the metastatic process. The tumor-specific immunosuppressive microenvironment serves an important function in tumor tolerance and escapes from immune surveillance leading to tumor progression. Therefore, identifying new drugs or foods that can enhance the tumor immune response is critical to develop improved cancer prevention methods and treatment. Recent studies have also indicated that curcumin can modulate tumor immune responses and remodel the tumor immunosuppressive microenvironment, indicating its potential in the immunotherapy of cancer (Mukherjee et al., 2018; Bahrami et al., 2019a; Pan et al., 2019). Curcumin has antimetastatic activities, modulating T cells, B cells, macrophages, neutrophils, NK cells, dendritic cells and production of cytokines and chemokines. In addition, recent studies have shown that curcumin exerts immunosuppressive effects (Shafabakhsh et al., 2019).

These findings showed that curcumin has crucial roles in the inhibition of angiogenesis and metastasis in GI cancers. Also, both the important events, including angiogenesis and inflammation, have been shown to contribute to metastatic formation and proliferation in GI cancers. Herein, we have summarized antimetastatic effects of curcumin in GI cancers.

## Metastasis

**FIGURE 1:**
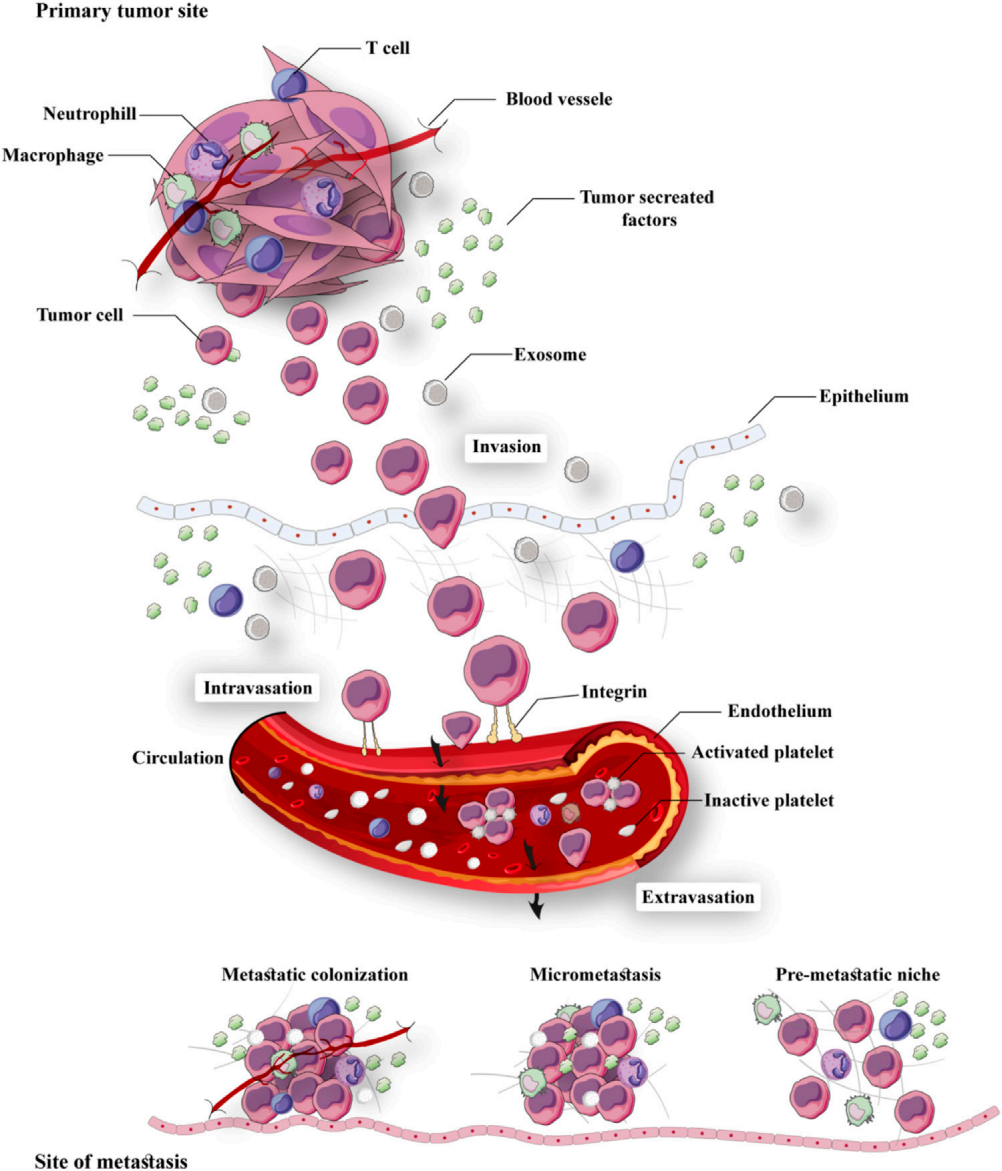
Summary of the metastatic cascade: The metastatic cascade is subdivided into multiple main steps, including invasion, intravasation, circulation, extravasation, and colonization.

**FIGURE 2:**
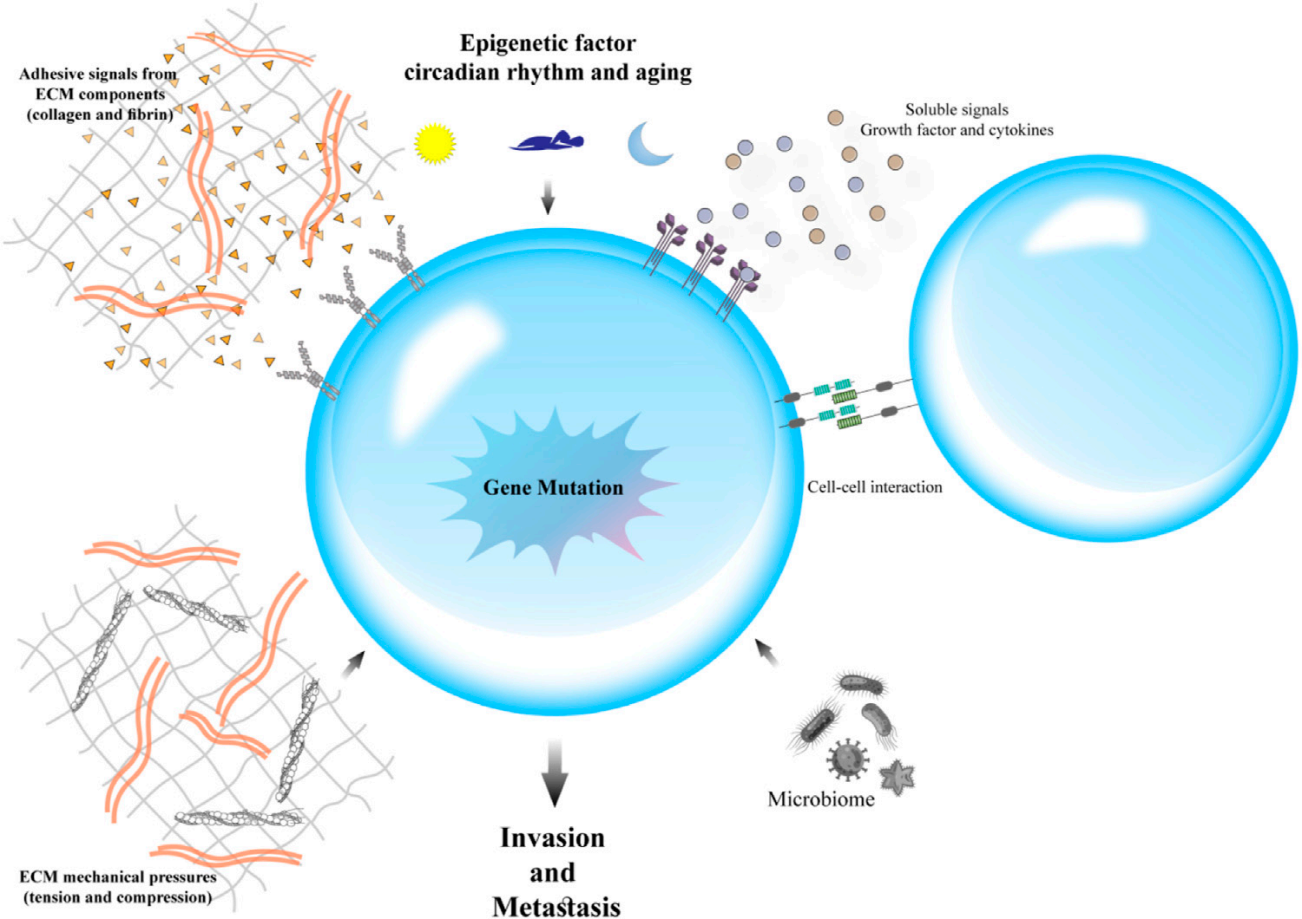
Factors involved in cancer metastasis: Several molecular and cellular factors contribute to invasion and metastasis activation, including epigenetic factors, adhesive signals of extracellular matrix (ECM) components, ECM mechanical pressures, cell–cell interactions, soluble signals, and the intratumoral microbiota.

“Metastasis” is an important process in which secondary tumors are developed in one of the organs that are separate from the original primary cancer site. Considering its considerable functions in the induction of failures in cancer management and increasing rate of mortality, apoptosis has been poorly understood currently. Although *in vivo* studies suggested that the percentage of metastases in melanoma cancer patients is <0.1% of tumor cells, but a more significant number of cancerous cells are released in blood circulation in other types of cancer (Luzzi et al., 1998). To metastases progression, tumoral cells must move from their primary tumor site, disperse in blood circulation, tolerate blood vessels' pressure, conform to new cellular settings in a secondary tumor location, and avoid immunological responses (Maitra, 2019; Massagué and Obenauf, 2016). Hanahan and Weinberg (2011) have declared that “invasion and metastasis activation” are signs of tumors. Therefore, metastasis as a major characteristic of cancer malignancies, accession to power from presentation of invasiveness features in surrounding tissue and homing on distant sites. Several factors such as tumor-secreted factors and exosomes dictate metastatic development (Figure 1). Unfortunately, metastasis represents the primary cause of death in a percentage of >90% of cancer patients (Steeg, 2006). Herein, comprehending connections of metastatic processes help us to recognize molecular and cellular targets for designing optimal therapies for suppressing or attenuating metastasis and consequent cancer progression. As mentioned earlier, cancer cell dissemination is the initial step of the metastases processes (Lambert et al., 2017). Chromosome segregation continuous errors during mitosis can lead to chromosomal instability, which is the potential cause in the induction of metastasis cascade (Figure 2). Numerous molecular and cellular factors have roles in invasion and metastasis activation, such as epigenetic factors, adhesive signals of extracellular matrix (ECM) components, ECM mechanical pressures, cell–cell interactions, soluble signals, and the intratumoral microbiota. Rupture of micronuclei via chromosome segregation errors leads to genomic DNA secretion into the cytosol. It functions as a cytosolic DNA-sensing pathways activator (cyclic GMP-AMP synthase–stimulator of interferon (IFN) genes) and finally activation of nuclear factor *?*-light-chain-enhancer B (NF-κB) signaling pathway (Bakhoum et al., 2018).

**FIGURE 3:**
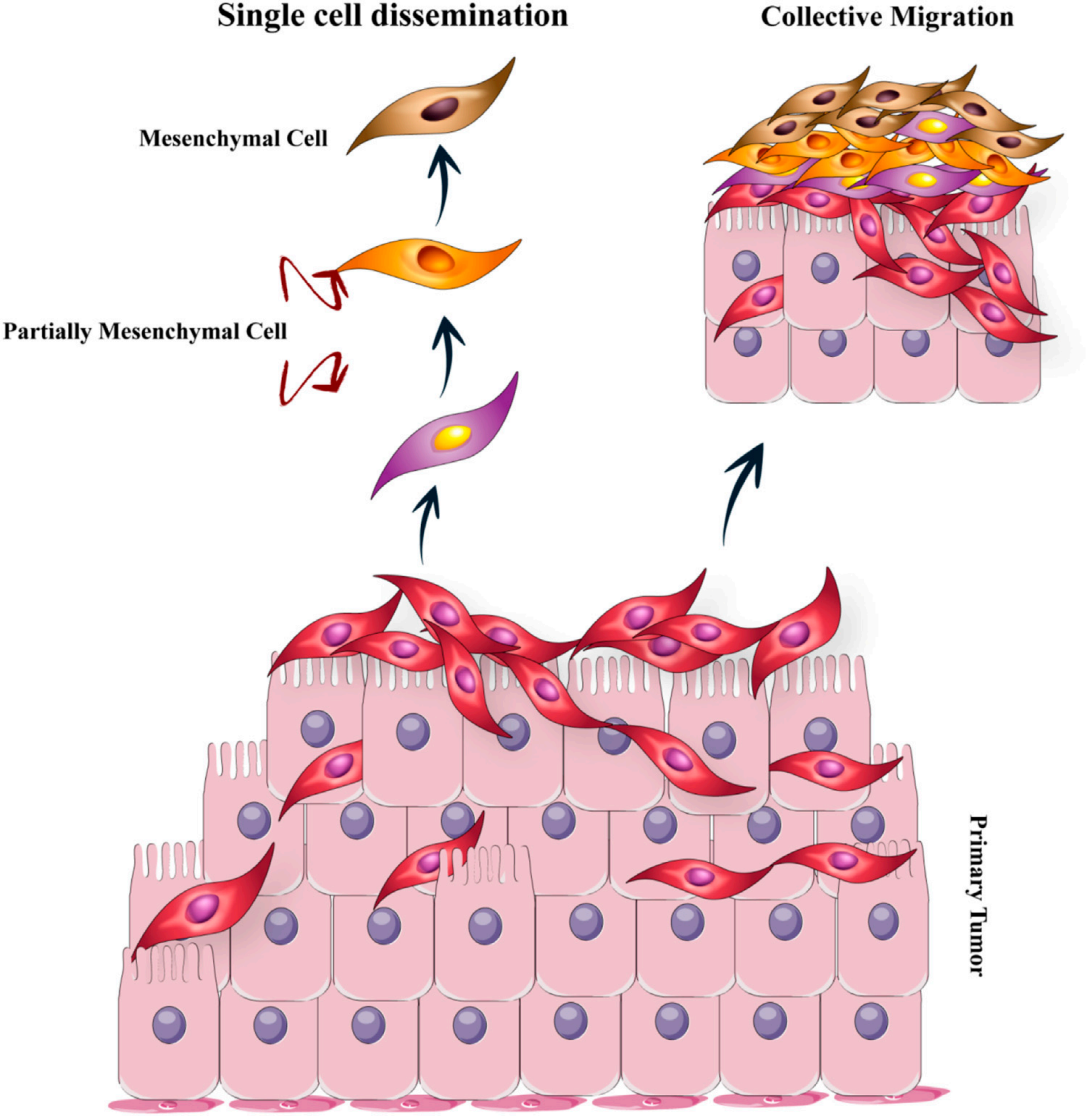
The epithelial–mesenchymal transition (EMT): The EMT program is a cell state that epithelial cells acquire mesenchymal cell properties and are capable of migration. There are two main invasion patterns of tumor cells: individual cell dissemination and collective cell migration.

Various studies have suggested that the primary disseminate nature of tumoral cells can be used as a potent determinant (Tabassum and Polyak, 2015; Gundem et al., 2015). An individual migration pattern of metastatic cancer cells was observed *in vivo* and *in vitro* investigations (Clark and Vignjevic, 2015). Inversely, in human cancer patients, the concerted action of a cluster of tumor cells is required for seeding (Cheung and Ewald, 2016). The ability of invasiveness, resistant stress, and dissemination of transformed epithelial cells develop in a trans-differentiation process called epithelial–mesenchymal transition (EMT) (Hanahan and Weinberg, 2011). Immotility and tight bounding together are some of the key features of epithelial cells. Also, epithelial cells are closely allied to the neighboring extracellular matrix (ECM) (Fouad and Aanei, 2017). Through governing reversible biochemical alterations, EMT accounts for permitting a specific epithelial cell for achieving a mesenchymal phenotype that admits significant epithelial–mesenchymal plasticity (Ye and Weinberg, 2015). Epithelial–mesenchymal plasticity is an essential specification for the development and metastasis of cancer cells (Figure 3). There are two main invasion patterns of tumor cells: single-cell dissemination and collective-cell migration. Current understanding of metastatic cell migration has relied primarily on studies on single-cell migration. However, the current paradigm focused on single-cell movements is shifting toward a dogma that collective migration is likely one of the primary modes of migration during metastasis of many solid tumors. Not surprisingly, the mechanics of collective migration differ significantly from single-cell movements (Lintz et al., 2017).

Moreover, it has been proved that all of the tumoral cells which originated from the primary site of the tumor have not to function in the contribution of metastasis development. It has been reported that an increased level of asparagine synthetase, which acts as a pivotal metabolic enzyme, is associated with metastasis progression in mouse-bearing breast cancer (Knott et al., 2018). Treatment with ʟ-asparaginase or dietary restriction-mediated decreasing in the level of asparagine has been reported to be an optimal strategy for reducing metastatic spread. Furthermore, recent studies have demonstrated that the availability of asparagine could lead to promoted EMT (Knott et al., 2018). Recently, a pseudo choice between full-mesenchymal phenotypes or full-epithelial in the EMT process has been rejected. At the same time, we have understood that EMT is a program with various transitional stages between the mesenchymal and epithelial phenotypes (Nieto et al., 2016). Various growth factors (Katsuno et al., 2013) and signaling cascades are governing the transition of the stage to each other in the EMT program (Craene and Berx, 2013). To that end, spontaneous EMT is shifting between various intermediate stages and intermediate phenotypes with extended invasive, metastatic, and differentiation features in primary tumor cells (Pastushenko et al., 2018). For observing better effectiveness in circulation and colonization at the secondary site and the consequent progression of metastases, tumor cells must have a combination of mesenchymal and epithelial phenotypes in EMT (Pastushenko et al., 2018). Numerous distinct and common transcriptional factors and signaling pathways regulate different cellular characteristics, chromatin landscapes, and gene expression signatures in various EMT stages (Pastushenko et al., 2018).

## Metastasis Induction

### Metastasis and Matrix Metalloproteinases

Degrading of basal membrane and extracellular matrix, which is caused by proteolytic agents such as matrix metalloproteinases (MMPs), can lead to peritumoral matrix destruction and subsequent invasive growth pattern (Brooks et al., 2010). To that end, MMP plays a crucial role in the invasion and metastases through entering the lymphatic system and blood circulation and then increasing the dissemination of tumor cells from the primary tumors into the enclosing tissues and secondary tumor sites.

In various *in vivo* and *in vitro* experimental studies, the upregulated expression level of MMPs has been correlated with significant growth and proliferation behavior of tumoral cells (Bachmeier et al., 2000a; Bachmeier et al., 2000b; Bachmeier et al., 2001; Murphy and Nagase, 2008). MMPs play pivotal functions in various tumor development features via affecting numerous biological processes. These features include activation of growth factor (Egeblad and Werb, 2002), tumor growth, induction of invasiveness, tumor-related inflammation, revascularization, and metastasis (Han et al., 2001; Philip et al., 2001). Hence, controlling expression levels of MMPs and suppression of its activation can be considered an optimal strategy for the prevention of cancer development.

Gene transcription is the first level that MMP expression inhibition initiates at that level. The promoter regions contribute to encoding the MMP-1, -2, -3, -7, -9, -12, and -13 genes, which are similar to NF-κB elements, carry a proximal activating-protein-1 (AP-1) binding site about 70 base pair 5′ for starting the transcription (Vincenti et al., 1998; Westermarck and Kähäri, 1999; Bond et al., 2001; Vincenti, 2001).

The properties of curcumin on the expression level of MMP and its functions have been investigated in different experimental studies in various kinds of inflammatory diseases and cancer models (Lin et al., 1998; Banerji et al., 2004; Swarnakar et al., 2005; Hong et al., 2006; Mitra et al., 2006; Su et al., 2006; Bachmeier et al., 2007; Shakibaei et al., 2007). By downregulating the expression of AP-1 and NF-κB, curcumin has been demonstrated to be a significant suppressor of MMPs synthesis (Bachmeier et al., 2007). Kim et al. reported that, through suppressing the activation of NF-κB and AP-1, curcumin inhibited the expression of MMP-9 and accompanied cell invasion mediated by12-O-tetradecanoylphorbol-13-acetate treatment (Kim et al., 2012a).

Bachmeier and colleagues demonstrated that the MMP-1 and MMP-2 mRNA expression and protein levels were considerably diminished after treatment with curcumin in breast cancer cells. In contrast, there was no considerable decrease in the expression levels of MMP-3 and -9 (Bachmeier et al., 2007). Furthermore, gelatinolytic activity has been evaluated by zymography, revealed that gelatinases MMP-2 and -9 proteolytic activities, down-regulated after treatment with curcumin.

An *in vitro* investigation on human fibrosarcoma cells demonstrated that the MMP-2 and MMP-9 expression levels were remarkably suppressed after treatment with three separate curcuminoids (demethoxycurcumin, bisdemethoxycurcumin, and curcumin). In comparison, there were no remarkable properties of the invasiveness of these cells and the urokinase plasmin activator (uPA) (Yodkeeree et al., 2008). To that end, in laryngeal squamous carcinoma cells, curcumin treatment induced significant down-regulatory impacts on the MMP-2, MT1-MMP expression, integrin receptors, and focal adhesion kinase (FAK), which led to a remarkable reduction in the invasiveness features of the tumoral cells. It is essential to state that the expression levels of MT1-MMP, MMP-2, integrin receptors, and FAK were similar to regulated expression levels after drug withdrawal (Mitra et al., 2006). Treatment with curcumin in human colon cancer cells caused a significant up and downregulation of MMP-9 and MMP-2, respectively. But any remarkable effect on the MMP-7 expression has not been observed, as evidenced by protein levels in Western blotting assay (Su et al., 2006). Curcumin treatment caused a noteworthy reduction in MMP-2 and MMP-9 expression, in conjunction with reduced cellular invasion *in vitro* in prostate cancer cells (DU-145). Besides, curcumin treatment diminished tumorigenicity in a xenograft model (Hong et al., 2006). Fifteen days of intervention with curcumin in a metastatic model of murine melanoma cells B16F10 remarkably decreased MMP-2 activity (Banerji et al., 2004). Besides, curcumin also reduced FAK and MT1-MMP expression. Results showed that even following 28 days, drug withdrawal could not return the MMP-2, MT1-MMP, and FAK expression to control levels. In the same study, curcumin caused a significant reduction in the level of invasiveness and migration and, reversely, promoted apoptosis *in vitro* (Philip and Kundu, 2003; Philip et al., 2004). In glioma cell lines, curcumin suppressed the TPA-mediated expression level of MMP-1, -3, -9, and -14-related mRNAs (Woo et al., 2005). On the other hand, following the curcumin intervention, the expression of numerous cell survival and proliferative inducer agents, such as cyclin D1, COX-2, IL-6, MMP-9, and Bcl-2, were significantly suppressed in the squamous carcinoma cells of the head and neck (Aggarwal et al., 2004). Following the curcumin treatment in a xenograft model of prostatic cancer, MMP-9 and MMP-2 expression remarkably decreased. Furthermore, a remarkable inhibitory effect on the invasion ability of the tumoral cells was observed *in vitro*. In this study, tumor volume, several metastatic nodules in the animal model, and the MP-2 and MMP-9 activity in the tumor-bearing site reduced significantly (Hong et al., 2006). Along with inhibition of ER downstream pS2 and TGFβ target genes, curcumin treatment in MCF-7 cell lines of breast cancer and estrogen presence significantly suppressed the estrogen receptor (ER) expression. By this study, curcumin treatment also showed significant down-regulatory effects on MMP-2 expression, while TIMP-1 (tissue inhibitor of metalloproteinase) expression was markedly upregulated after curcumin treatment (Shao et al., 2002).

Besides, different studies also have evaluated the properties of curcumin on the invasiveness of lung cancer models: through suppression of MMP-2, -9, and VEGF expression, curcumin significantly decreased migration features and invasiveness of A549 cell line in a time-dose-dependent manner (Lin et al., 2009). The above-mentioned effects were also observed in an *in vivo* model by affecting the expression of the 801D cell. Inhibition of the Rac-dependent pathway by curcumin caused a remarkable decrease in the MMP-2 and -9 expression levels (Chen et al., 2014).

Overall, considering slight differences in the efficiency of curcumin in the inhibition of MMPs in some cell lines or studies, it seems that curcumin has significant modulatory effects in virtually all of MMPs. These minor discrepancies may result from slight differences in curcumin-related responsivity and partial resistance of some cell lines (Bachmeier et al., 2009). Furthermore, it is evident that curcumin considerably plays pivotal functions in inhibiting degradation in some of the extracellular matrix and basal membrane components. These effects are due to its potential action in regulating MMP/TIMP activity and expression, which was led to the significant impacts on the reversion of tumor invasion and growth.

### Metastasis and RAF/MEK/RAS/ERK Signaling Pathway

**FIGURE 4:**
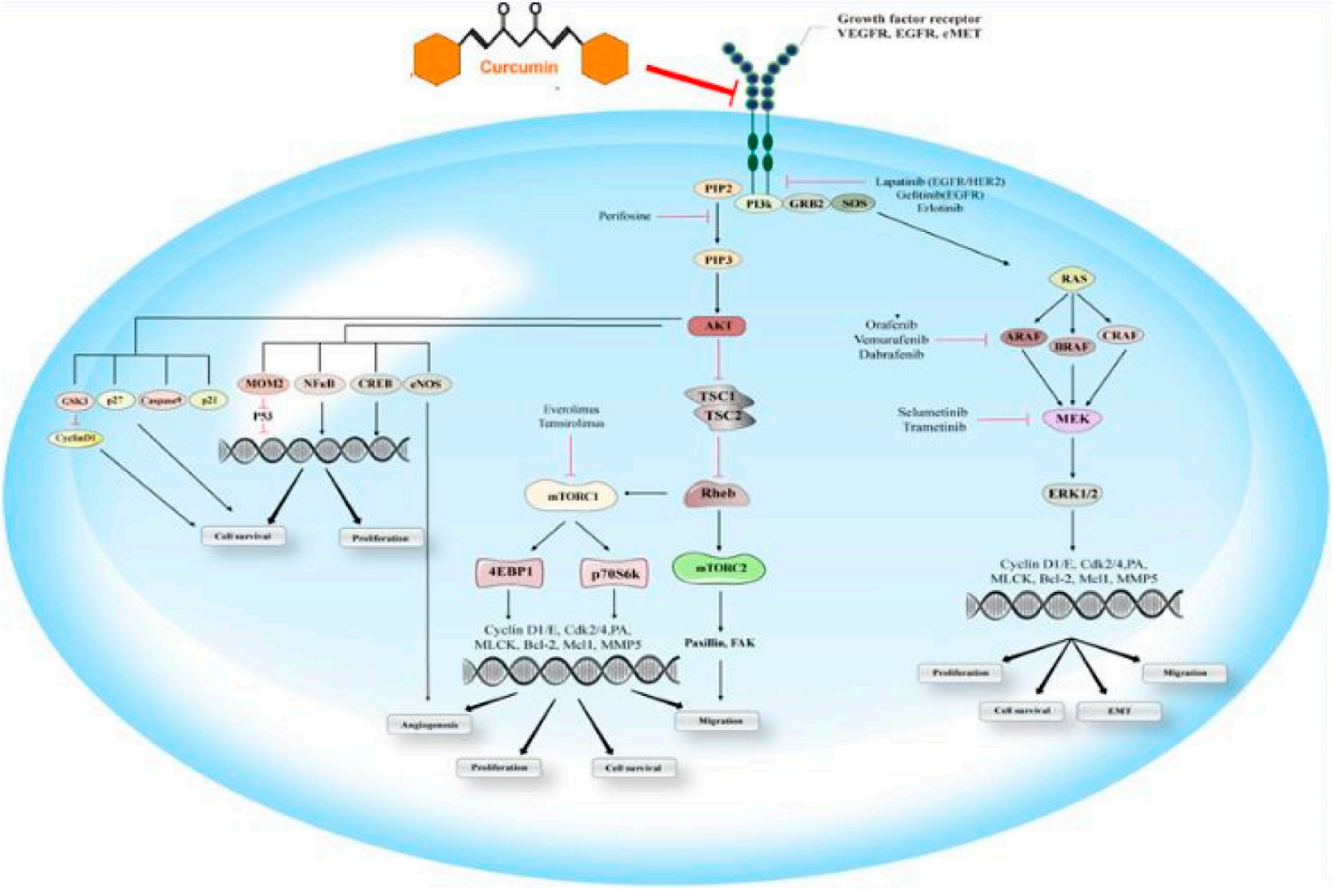
**EGFR signaling pathway:** This schematic indicates the essential components of the RAS/RAF/MEK/ERK and PI3K/Akt/mTOR signaling pathways and their efficacy on the cascade of metastasis. EGFR, an activated receptor tyrosine kinase, triggers both cascades that result in the stimulation of Akt, mTOR-1/-2, and ERK as the downstream effectors. Some drug agents target these signaling pathways, including panitumumab, vemurafenib, dabrafenib, cetuximab, trastuzumab, and gefitinib, sorafenib, selumetinib, lapatinib, temsirolimus, erlotinib, perifosine, trametinib, everolimus. Curumin is able to inhibit EGFR signaling.

The RAF/MEK/RAS/ERK signaling pathway is the primary modulator of different cellular processes. This signaling pathway contributes to the modulation of cell differentiation, proliferation, survival, and motility. It provides the transduction of signals from the cell surface to the nucleus and cytoplasm as well. Moreover, abnormal regulation of this signaling pathway is a potential reason for the initiation and progression of tumoral cells. Cell surface receptors-mediated activation of small GTPase rat sarcoma oncogene (RAS) homolog leads to binding binds and the consequent rapid activation of accelerated fibrosarcoma kinase (RAF). H-, K-, and N-RAS have been considered the most related members of the RAS family, clinically. RAF has three isoforms, including a-, b-, c-RAF, considering the major role b-RAF in tumorigenesis. RAF contributes to phosphorylation of MAPK and ERK kinase (MEK), which for its parts has crucial roles in the mitogen-activated protein kinases (MAPKs) activation. The primary MAPKs members have been reported as extracellular signal-regulated kinases (ERK) 1 and 2. Considering their important roles, ERK-1/-2 with a variety of substrates also have been considered major effectors of this pathway (Figure 4).

Activating RAS or b-RAF mutations has been significantly associated with higher RAS/RAF/MEK/ERK signaling activation. Moreover, various studies have demonstrated that K-RAS is the principal mutated RAS gene in the pancreas, lung, and colon adenocarcinoma samples (Bos, 1989; Campbell and Der, 2004). Besides, some of the receptor tyrosine kinases, such as EGFR, were correlated to the activation of RAS. Mutational activation or overexpressed level of EGFR has been associated with lower cancer prognosis. It is also associated with upregulation of signaling pathways, including RAS/RAF/MEK/ERK, PI3K/Akt/mTOR, protein kinase C (PKC), phospholipase D signal transducer, and activator of transcription (STAT) (Roberts and Der, 2007). Curcumin has been shown to exhibit anticancer effects by interfering with signaling pathways associated with the initiation, promotion, and progression of multistage carcinogenesis. Curcumin inhibits the ERK signaling pathway, thus blocking the cell cycle and downregulates the expression of Bcl-2, concomitantly inhibiting cell proliferation and inducing apoptosis (Zhu et al., 2016).

### Metastasis and Wnt Signaling Pathway

**FIGURE 5:**
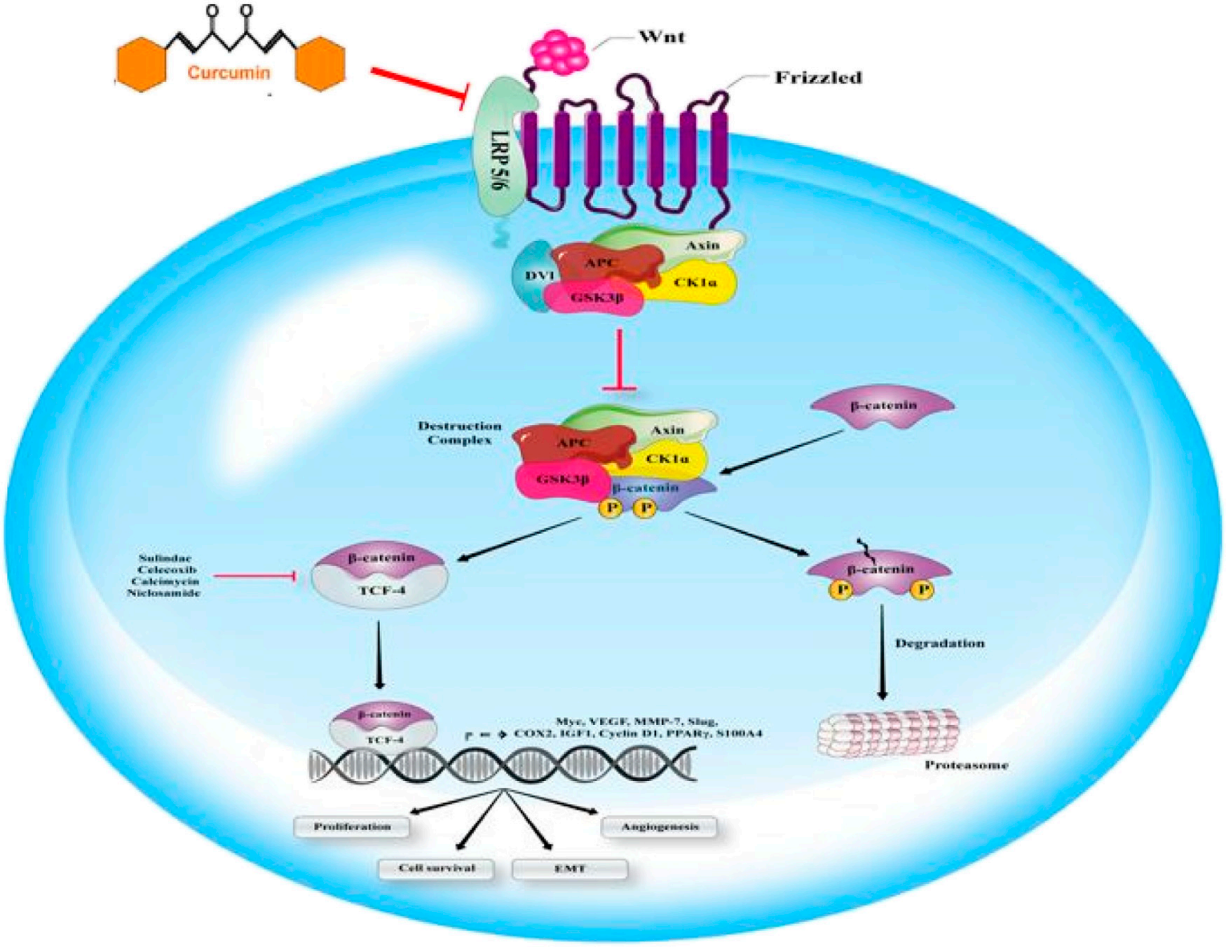
**Wnt signaling can affect the cascade of metastasis.** Wnt ligand binding to the multipass receptor leads to inhibiting demolition complex and the nucleic accumulation of β-catenin, involved in Wnt gene expression. Some of the antibiotic calcimycin, NSAIDs such as celecoxib or sulindac, and the anthelminthic niclosamide play a significant role in β-catenin-induced gene transcription targeting. Curumin is able to inhibit Wnt signaling.

Wnt signaling pathway plays pivotal functions regulating cell polarity, cell proliferation, and cell fate determination during embryonic development. It has been shown that in the absence of the Wnt signaling pathway, the multiprotein complex has the responsibility of regulating intracellular β-catenin levels. Investigations have displayed that adenomatous polyposis coli protein (APC) as a tumor suppressor, casein kinase 1 (CK1), glycogen synthase kinase-3β (GSK3β), and the scaffolding protein Axin are involved in the multiprotein complex structure. It has been reported that Cytoplasmic β-catenin is an essential target for induction of ubiquitination and proteasomal degradation by the destruction of complex-mediated binding and phosphorylating. As this elimination process continues, β-catenin cannot reach the nucleus any longer. Another important family of transcription factors is the DNA-bound T-cell factor/lymphoid enhancer factor (TCF/LEF), which has been suggested to be one of the principal regulators of β-catenin gene expression. Lacking the nuclear β-catenin, through inactivating Groucho/TLE, as transcriptional corepressors by binding to them, TCF/LEF contributes to suppressing gene expression. Wnt has a crucial role in Wnt/β-catenin pathway activation by binding its ligands to its co-receptor–low-density lipoprotein receptor-related proteins (LRP)—and also the Frizzled receptor. As a result, these receptors will be activated. Activation of Wnt/β-catenin pathway significantly hinders the destruction complex, and finally can lead to enhanced aggregation and nucleic translocation of β-catenin. It has been proved that, through binding to TCF/LEF and accompanied inactivation of them, and via dislocating the Groucho/TLE, transcriptional co-repressors, β-catenin activates Wnt target gene expression in the nucleus (Figure 5) (Daniels and Weis, 2005; Fodde and Brabletz, 2007). Curcumin inhibits tumor epithelial–mesenchymal transition by downregulating the Wnt signaling pathway and upregulating NKD2 expression in cancer cells (Zhang et al., 2016). Furthermore, curcumin suppresses cancer cell migration by inhibiting the Wnt signaling pathway (Kim et al., 2013).

## Curcumin and Metastasis in Gastrointestinal

### Regulation of Metastasis in Pancreatic Cancer by Curcumin

Curcumin-mediated effects on metastasis in pancreatic cancer cells are associated with their properties on various heterotypic cells—these cells, including endothelial cells, macrophages, cancer-associated fibroblasts (CAFs). CAFs have been implicated in different malignancies, such as pancreatic ductal adenocarcinoma (PDAC), breast cancer, and gastric carcinoma (Kasashima et al., 2014; Sun et al., 2014). By targeting numerous cellular events in cancerous cells such as cell viability, tumor growth, metastasis, revascularization, immune surveillance, and CAFs, curcumin plays a crucial role in regulating tumor behavior (Lunardi et al., 2014). It has been reported that the expression level of α-smooth muscle actin (α-SMA) and vimentin is significantly correlated with the process of attaining an activated phenotype by CAFs. Besides, for transforming into fibroblast-like cells, tumoral cells can potentially make use of EMT (Kalluri and Zeisberg, 2006). EMT has been introduced to be a transcriptional and epigenetic process in which some of the mesenchymal features, including enhanced motility and decreased cellular junctions, can be acquired by epithelial cells, which have important roles in the dissemination of primary tumor sites and cancer seeding at distant organs (Thiery, 2002; Friedl, 2004). In addition to CAFs capabilities for affecting the EMT of cancer cells, it has been proven that the expression of fibroblast-like cancer cells is considerably associated with higher invasion and metastatic features. Reduced expression of E-cadherin, which has been associated with tumor progression and development, is an important sign of EMT (Cowin et al., 2005; Christofori, 2006; Francí et al., 2006).

Wang and colleagues theorized that CAFs might be a possible target of curcumin for inhibiting pancreatic cancer cells’ metastases (Wang et al., 2017). Their results have shown that curcumin significantly suppressed the CAF-induced promotion of metastasis and migration abilities in pancreatic cancer cells. Overall, by decreasing the mesenchymal features of CAFs, data provided substantial evidence that curcumin suppressed pancreatic cancer cells’ metastasis and migration characteristics. It resulted in the reversed mediation of EMT phenotypes in pancreatic cancer cells (Wang et al., 2017).

Hypoxia is low oxygen tension traditionally found in solid tumors, which is considerably associated with decreased prognosis in cancer patients (Lei et al., 2013; Chang and Erler, 2014). Results have shown that tumor hypoxia-induced behavior of tumoral cells is significantly correlated with promoted invasion, angiogenesis, and metastasis in distant tumor sites (Chang and Erler, 2014). Hypoxia-inducible factor-1 (HIF-1) is a member of the primary helix-loop-helix-periodic acid-Schiff domain transcription factor family and has been implicated in several types of solid tumors, such as pancreatic cancer. HIF-1haskey functions in the mediation of hypoxia-induced cellular responses (Lei et al., 2013). In their study, Sun et al. (2013) demonstrated that via inhibiting the Hh signaling pathway, the progression of pancreatic cancer was diminished after curcumin intervention. As a crucial pathway in tumorigenesis, the Hh signaling pathway has been shown to be implicated in pancreatic cancer samples while it is quiescent in adult pancreas cells in a normal situation (Lei et al., 2013). In a recent study, Lei et al. have shown that hypoxia-mediated invasiveness and occurrence of EMT is closely associated with the Hedgehog signaling pathway (Lei et al., 2013). Cao and colleagues hypothesized that curcumin is a prominent inhibitor for preventing hypoxia-induced invasiveness, proliferation, metastasis, and EMT progression in pancreatic cancer. Besides, they have been shown that curcumin strongly suppressed hypoxia-induced activation of Hedgehog signaling pathway. It suggested that curcumin may be a potential novel treatment for optimizing current therapeutic treatments against pancreatic cancer (Cao et al., 2016a). The results reported that in addition to its properties against hypoxia-mediated activation of Hedgehog signaling pathway, cell migration, proliferation, and invasiveness of pancreatic cancer, curcumin also mediated the EMT-related agent’s expression including vimentin, E-cadherin, and N-cadherin. Taken together, Cao and colleagues have demonstrated that via suppressing the Hh signaling pathway, curcumin plays critical roles for the inhibition of hypoxia-mediated metastasis of pancreatic cancer (Cao et al., 2016a).

Hydrogen peroxide (H2O2) and superoxide anion as reactive oxygen species (ROS) are some chemically oxygen-derived reactive agents. They are generated by the mitochondrial respiratory chain. In various studies, the state of intracellular redox has been correlated with cellular signaling transduction, and regulation of multiple intracellular events (Lee and Kang, 2013). Despite their significant roles for suppressing cancer cells, via affecting cell viability, proliferation, invasion, and metastasis, sublethal concentrations production of ROS has been associated with tumor progression (Nishikawa et al., 2009). For example, Nishikawa et al. showed that sublethal concentrations of H2O2 (0–200 *µ*M) resulted in pancreatic cancer development in a dose-dependent manner. In contrast, > 200 *µ*M concentrations of H2O2 were cytotoxic for cancerous cells (Li et al., 2015).

Recently, a study demonstrated that curcumin had strong protective properties against the EMT process in the prostate cancer cells. Curcumin abrogated CAF-induced invasion and EMT, and inhibited ROS production and CXCR4 and IL-6 receptor expression in prostate cancer cells. These effects were mediated by inhibiting MAOA/mTOR/HIF-1α signaling. It was found that this protective effect correlated with MAOA/mTOR/HIF-1α signaling pathway-induced inhibition of CAFs-mediated ROS generation (Du et al., 2015). As crucial downstream signaling cascades of ROSs, MAPK signaling pathways have been demonstrated to participate in tumor progression (Wu et al., 2008). P38 MAPK, extracellular signal-regulated kinase (ERK), and c-jun NH-2 terminal kinase (JNK) are the most critical MAPK family members. Li et al. have declared that via activating p38 MAPK and ERK signaling pathways, moderate amounts of H2O2 are associated with promoted pancreatic cancer metastasis and invasion (Li et al., 2015).

**FIGURE 6:**
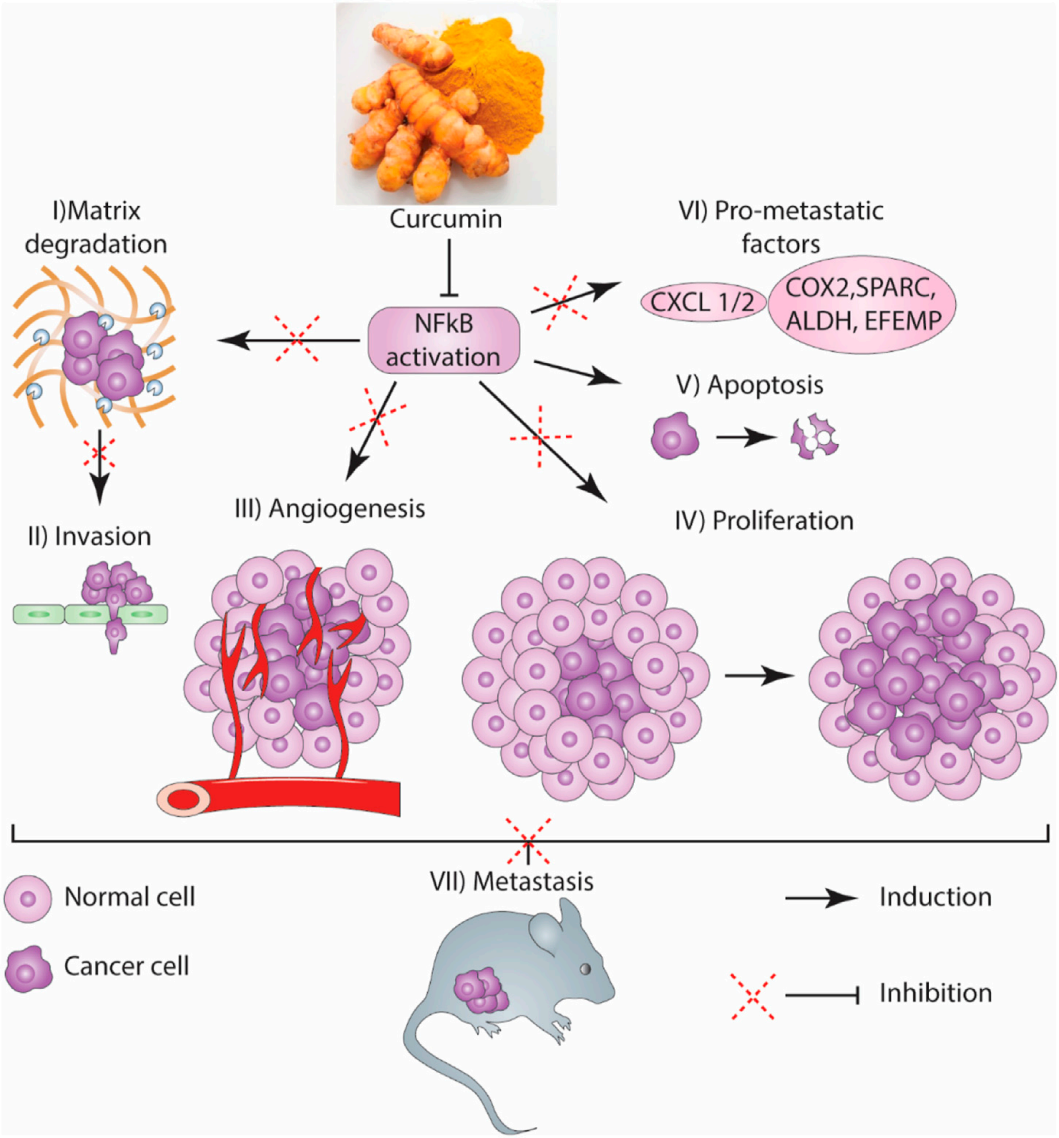
A schema of the antimetastatic effects of curcumin in GI cancers.

Cao et al. showed that curcumin intervention in Panc-1 and BxPC-3 pancreatic cancer cells reversely regulated the cancer invasion, migration, and MMP-2 expression. Besides, H2O2-mediated upregulation of phosphorylated ERK and phosphorylated NF-κB was decreased after NAC, curcumin, and PD 98059 treatment (an ERK inhibitor). Considering this valuable information, it can be concluded that curcumin inhibited pancreatic cancer cell migration and invasiveness via inhibiting the ROS/ERK/NF-κB signaling pathway. Furthermore, this study suggested that curcumin application might be a possible drug target for upregulating pancreatic cancer migration (Cao et al., 2016b). Table 1 lists various studies on antimetastatic effects in pancreatic cancer. Figure 6 illustrates the effects of curcumin on metastasis in GI cancers.

**TABLE 1:** Curcumin and metastasis in pancreatic cancer.

Type of curcumin	Dose	Target	Model	Cell line	Results	Ref
Curcumin	5 and10 µM	E-cadherin and Vimentin	*In vitro*, In vivo	Capan1 and Panc1	- Blocked migration and metastasis	Wang et al. (2017)
Irinotecan and curcumin in ultra-small PEGylated NDs, curcumin	15 mg/kg	Kras and Trp53	In silico, In vitro, and In vivo	AsPC-1 and PANC-1	- Antitumor efficacy	Madamsetty et al. (2019)
					- Downregulation of modulator of the tumor microenvironment	
Curcumin	20 µM	E-cadherin, N-cadherin, vimentin, and Hh signaling-related factors (SHH, SMO, GLI1)	*In vitro*	Panc-1	- Inhibition of cell proliferation, migration, and invasion	Cao et al. (2016a)
Curcumin	5, 10, 20, and 40 µM	(MMP)-2, MMP-9,p-ERK, and p-NF-κB	*In vitro*	BxPC-3and Panc-1	- Inhibition of cell invasion and migration	Cao et al. (2016b)
Curcumin, EF31, and UBS109	10 μM curcumin, 750 nM EF31, and 250 nM UBS109 in MIAPaCa-2 cells. 20 μM curcumin, 1.25 μM EF31, and 250 nMUBS109 in PANC-1 cells (	TGFβ, angiopoietin 1, angiopoietin 2, HIF-1α, Hsp90, COX-2, VEGF, and NF-κB	*In vitro*	MIA PaCa-2 and PANC-1	- Antiangiogenic activities	Nagaraju et al. (2015)
					- Downregulation of HIF-1α, Hsp90, COX-2, and VEGF	
Curcumin	20 μM	E-cadherin, N-cadherin, vimentin, and PI3K/Akt/NF-κB signaling pathway	*In vitro*	BxPC-3 and Panc-1	- Inhibition of epithelial-to-mesenchymal transition via the PI3K/Akt/NF-κB pathway	Li et al. (2018a)
Curcumin loaded chitosan/PEG blended PLGA nanoparticles	10 mg	Bcl2, Bax, PARP, and Caspase-3	*In vitro*	PANC-1 and mia Paca-2	- Enhanced antimigratory, anti-invasive, and apoptosis effect	Arya et al. (2018)
Curcumin	2.5, 5, 15, 30, and 50 μM	Caspase-3 and Caspase-9	*In vitro*	BxPC-3 and Panc-1	- Curcumin and garcinol in combination exhibit a high level of synergism, with enhanced bioactivity	Parasramka and Gupta (2012)
Curcumin	0–100 μM	TNFR, caspase-8, caspase-3, BID, Bax, NFκB, NDRG 1, and BCL2L10	*In vitro*	BxPC-3 and MiaPaCa-2	- Upregulation of the extrinsic apoptotic pathway	Youns and Fathy (2013)
Curcumin	10, 20, and 30 μmol/ml	Shh, GLI1, E-cadherin, and vimentin	*In vitro*	PANC-1	- Reversed the epithelial–mesenchymal transition of pancreatic cancer cells by inhibiting the hedgehog signaling pathway	Sun et al. (2013)
Polymeric nanoparticle-encapsulated curcumin	150 μL nanocurcumin and 25 mg/kg curcumin	NFκB, MMP-9, and cyclin D1	*In vivo*	-	- Blocked tumor growth and metastasis	Bisht et al. (2010)
Curcumin	50, 100, or 200 nM	NF-κB, caspase-3, and -7	*In vitro*	Panc-1, BxPC-3, and MIA PaCa-2	- Inhibited cell viability/survival, robustly activated caspase-3/7 activity, and subsequent cell death	Veeraraghavan et al. (2011)
CDF (a synthetic curcumin-derived analogue)	0.5–2 µM	PTEN, MT1-MMP, miR-200 family, and β-actin	*In vitro*	AsPC-1, BxPC-3, COLO-357, MIAPaCa-2, MIAPaCa-GR, and PANC-1	- Re-expression of miR-200c	Soubani et al. (2012)
					- Downregulated the expression of MT1-MMP was	
Curcumin	?	ERK, NF-kB, E-cadherin, vimentin, MMP-9, and IL-6	*In vitro*	BxPC-3 and Panc-1	- Inhibition of growth and metastasis	Mardani et al. (2020)
CDF (a synthetic curcumin-derived analogue)	0.5 µM	VEGF, IL-6, Oct4, EZH2, miR-21, miR-210 u, Nanog, EZH2, and HIF1-α	*In vitro* and In vivo	AsPC-1 and MiaPaCa-2	- Attenuated the aggressiveness of cancer cells through decreasing the expression of VEGF, IL-6, and miR-21	Bao et al. (2012)

### Regulation of Metastasis in Gastric Cancer by Curcumin

It has been demonstrated that high mobility group box 1 (HMGB1), as an essential nuclear and extracellular protein, is a crucial mediator in various pathologic and physiologic situations such as inflammation, immune response, and cancer. HMGB1 is also a diagnostic biomarker for the early stages of gastric cancer (Chung et al., 2009). Curcumin treatment in human endothelial cells caused considerable down-regulatory properties on the expression of the cell surface receptor of HMGB1 (Kim et al., 2011). Studies have reported that HMGB1 expression is highly increased in esophageal squamous cell carcinoma (ESCC). HMGB1 has a crucial role in promoting lymphangiogenesis by regulating vascular endothelial growth factors (VEGF)-C. Lymph node metastasis has been considered one of the critical determinants of progression in gastric cancer patients. Studies have declared that HMGB1 expression is associated with tumor lymphangiogenesis and new lymphatic vessel formation (Tawada et al., 2012). Curcumin treatment resulted in VEGF receptors’ downregulation, including (VEGFR-2/3) and lymphangiogenic VEGF-C (Da et al., 2015). HMGB1 and VEGF-D are involved in tumor lymphangiogenesis, and VEGF-C and VEGF-D are also essential members of the VEGF family.

In a recent study, Da and colleagues hypothesized that by suppression of HMGB1/VEGF-D signaling, curcumin might have potential anti-lymphangiogenesis properties. Therefore, it can be applied as a potential treatment in patients with gastric cancer (Da et al., 2019). Results have demonstrated that curcumin treatment in AGS and SGC-7901 gastric cancer cells caused a significant cell viability reduction through activating caspase-3. In contrast, it resulted in an apoptosis increase in a concentration-dependent manner. Besides, curcumin intervention remarkably eliminated the HMGB1 and VEGF-D expression levels. Considering their findings, they have suggested that curcumin might be an anti-lymphangiogenesis agent in the treatment of gastric cancer by inhibiting HMGB1/VEGF-D signaling (Da et al., 2019).

Late-stage diagnosis, which is seen in most gastric cancer patients, causes poor prognosis and therapeutic outcome. This problem results in tumor cell dissemination into circulation and distant organs as circulating tumor cells (CTCs), which leads to cancer seeding, and formation of a distal tumor, especially in hepatic tissue (Saito et al., 2013; Hatakeyama et al., 2015; Xia et al., 2015). In addition to their presence in blood circulation, CTCs have been identified with a number of specific cell surface markers. Among them, CXCR4, which is known as the stromal cell-derived factor-1 (SDF-1) receptor, has been characterized by different cancer types (Reckamp et al., 2009; Franco et al., 2012; Mego et al., 2016). In their previous study, Zhu et al. have declared that CD90^+^ and CXCR4+ hepatocellular carcinoma (HCC) cells may be CTCs. Therefore, selective inhibition of CD90^+^ and CXCR4+ cells, resulting in reduced cancer metastasis, may provide significant improvements in the current therapeutic approaches in HCC patients (Zhu et al., 2015).

Gu et al. have evaluated the antimetastatic capabilities of curcumin in the reduction of tumor formation in liver in mice-bearing proximal gastric carcinoma (PGC) (Gu et al., 2019). Results showed that formation of hepatic tumor and presence of CTCs were considerably reduced after curcumin treatment. Furthermore, treatment with curcumin in PGCs *in vivo* and *in vitro* downregulated the CXCR4 expression, and hence by inhibiting the SDF-1/CXCR4 signaling pathway, suppressed metastasis of PGC (Gu et al., 2019).

By suppressing the activity of MMP-2, curcumin administration has been associated with inhibiting cancer progression (Mitra et al., 2006; Lee et al., 2015a; Liu et al., 2017). Moreover, due to its low solubility, bioavailability and efficacy of curcumin is decreased. Various *in vivo* investigations have demonstrated that induction of structural modifications in the β-diketone moiety, aromatic rings, or the flanking double bonds coupled with the β-diketone moiety of curcumin may be an optimal strategy for improving its bioavailability and anticancer activities. Selected CH-5 (4,4′-[(2-Oxo-1,3-cyclohexanediylidene)-di(E) methylylidene]dibenzonitrile) and monoketone curcumin analogs have been screened to be evaluated for their anticancer properties in numerous tumor cell lines (Lima et al., 2018).

Silva and colleagues investigated the antimetastatic properties of CH-5 in human HGC-27 gastric cancer cell line (Silva et al., 2018). Initially, they have observed cell viability was decreased, while apoptosis was increased in a dose-dependent way. Additionally, through decreased expression and collagenase activity of MMP-2, a remarkable decrease in the invasiveness and migration of HGC-27 cells was observed after CH-5 treatment. Herein, via the upregulation of apoptosis and the downregulation of invasion and migration, CH-5 treatment in gastric cancer cells showed anticancer properties. It implies that the application of CH-5 molecule can be considered an optimal antimetastatic medicine for the prevention of gastric cancer development (Silva et al., 2018). Table 2 lists various studies on antimetastatic effects of in gastric cancer.

**TABLE 2:** Curcumin inhibits gastric cancer metastases.

Type of curcumin	Dose	Target	Model	Cell line	Sample size	Results	Ref
Curcumin	10–50 μM	Caspase-3, VEGF-D, and HMGB1	*In vitro*	AGS and SGC-7901	-	- Anti-lymphangiogenesis effects	Da et al. (2019)
						- Decreased cell viability and apoptosis through the activation of caspase-3	
Curcumin and liposomal curcumin	0.5 μmol/L in vitro and 10 mg/kg in vivo	CXCR4, GAPDH, and SDF1	*In vitro*, In vivo	PGCs	30	- Inhibited metastasis through reducing circulating tumor cells	Gu et al. (2019)
Curcumin-loaded nanoemulsion and curcumin	12.5 μM	NA	*In vitro*, In vivo	AGS	10	- Prevent tumor reincidence and metastasis	Guerrero et al. (2018)
CH-5	0, 2.5, 5, 10, 20, 40, and 50 µM	Caspase-3, MMP-2	*In vitro*	HGC-27	-	- Suppressed proliferation, migration, and invasion	Silva et al. (2018)
Curcumin	40, 80, and 160 mg/kg per day	VEGFR-3, Prox-1, podoplanin, and LYVE-1	*In vitro*, In vivo	SGC7901	10	- Suppressed lymphatic vessel density	Da et al. (2015)
B19	5, 10, or 20 μM	TrxR1	*In vitro*, In vivo	SGC-7901, BGC-823, and KATO III	-	- Induced ROS-dependent apoptosis and cell cycle arrest	Chen et al. (2016)
Hydrazinocurcumin (CTK7A)	100 µM	p300, Hif1α, Noxa, Twist1, caspase-3, caspase-9, Cytochrome *c*, N-cadherin, E-cadherin, p-p38 MAPK, p- ERK, p-phospho JNK, GAPDH, and Cox IV	*In vitro*	GCCs AGS, MKN 45, KATO III	-	- Reduced invasiveness of cancer cells and induced apoptosis	Rath et al. (2017)

### Regulation of Metastasis in Colorectal Cancer by Curcumin

*Cancer* stem cells (CSCs) are a small subpopulation of immortal cells. They are essential agents in chemoresistance, tumor invasion, and relapse. CSCs have crucial functions in metastasis induction, which is considered the leading factor of cancer-related mortalities (Muñoz et al., 2012). Numerous studies have shown that curcumin targeted Wnt/β-catenin, Notch, and Sonic Hedgehog (SHH) signaling pathways. These are involved in the self-renewal of tumoral cells, targeting CSCs to prevent cancer invasion (Takebe et al., 2015). Some investigators believe the anti-inflammatory properties of curcumin are additionally engaged in its antimetastatic characteristics.

It has been demonstrated that overexpression of insulin-like growth factor-1 (IGF-1) and insulin is an essential agent in cancer progression and metastasis (Adachi et al., 2002; Sarfstein et al., 2010; Liu et al., 2011; Orrù and Nigro, 2017). There is a lot of resemblance in action between insulin and IGF-1 signaling; herein, there is a possibility of recruitment of each other’s receptors by their ligands (Panda et al., 2013). Besides, through affecting similar mechanisms, both these receptors play crucial roles in the activation of similar intracellular pathways (Han et al., 2017). Inhibition of IGF-1 in some specific cancer types, such as pancreatic carcinomas, led to decreased cancer growth and metastasis. In contrast, the role of insulin receptor downregulation in the suppression of cancer development has not yet been fully understood (Subramani et al., 2014). Larsson and colleagues demonstrated that hyperinsulinemia is positively associated with progression of colorectal cancer in diabetic patients (Larsson et al., 2005). They also reported that through modulating mitogen-activated protein kinase (MAPK) and IRS1/phosphoinositide 3-kinase (PI3K)/Akt signaling pathways, insulin significantly increases the mRNA expression of (MMP-2) in HCT-116 human colorectal cells (Larsson et al., 2005).

Various investigations have suggested that anti-inflammatory properties of curcumin are involved into its inhibitory effects on migration of tumoral cells. Insulin and IGF-1 receptors and signaling pathways were regulated by curcumin. As an essential pathway, insulin signaling participates in tumor progression and initiation. To that end, Hosseini and colleagues hypothesized that via downregulating insulin and IGF-1 receptors, curcumin could show its antimetastatic properties (Hosseini et al., 2019). In their study, the effects of curcumin treatment were measured in 5-fluorouracil (5-FU)-treated viable resistant SW480 colorectal cells. Their results showed that the expression of avian myelocytomatosis virus oncogene cellular homolog (MYC), IGF-1 receptors, and insulin was remarkably decreased after treatment with curcumin. Therefore, insulin and IGF-1 receptors downregulation in chemoresistant colorectal cancer cells caused a greater reduction in the migration and proliferation of tumoral cells (Hosseini et al., 2019).

Application of curcumin in combination with different medicines including 5-FU (Toden et al., 2015), to lfenamic acid (Sankpal et al., 2016), and ulinastatin (Shen et al., 2014) can make its utilization more effective, reverse chemoresistance, inhibit cell growth, and metastasis. More than 300 numerous constituents, including free arabinose (1%), curcumin (3–5%), essential oil (2–7%), and acid glycans named ukonan, were extracted from turmeric, which is known as the significant source of curcumin (Kim et al., 2012b). Moreover, various studies demonstrated that besides curcumin, different components in turmeric have been introduced to be more potent for displaying antitumor activities, and can be used as potential enhancer of curcumin bioavailability (Anand et al., 2007; Kim et al., 2012b). Yue et al. demonstrated that the accumulation of curcumin within colonic cells was increased in the presence of turmerones in the extract of turmeric (Yue et al., 2012). Furthermore, curcumin plus turmerones or curcumin in the extract of turmeric can promote the antitumor properties in the colorectal cancer model *in vivo* (Yue et al., 2016). Thus far, a few investigations evaluated the turmeric extract antimetastatic properties on colorectal cancer. According to Kim et al., CXC motif receptor 4 (CXCR4) expression, as a chemokine receptor that has a role in metastasis *in vitro*, was inhibited after turmeric extract treatment (Kim et al., 2012b).

Using an aggressive orthotopic CRC model with spontaneous metastasis, Li et al. evaluated antimetastatic and antitumor properties of turmeric extract and first-line chemotherapeutics (FOLFOX) (134). Their results showed turmeric extract significantly induced a cytotoxic effect, suppressed colony formation, reduced cell motility, migration, and EMT in murine colorectal cancer cells. Turmeric extract showed all these functions using various signaling pathways such as cofilin, ERK, FAK/p-Src, STAT3, and AKT. Besides, *in vivo* treatment with turmeric extract (200 mg/kg) caused a great reduction burden of a colon tumor, and suppressed hepatic and lung metastasis. Also, turmeric extract treatment significantly promoted immune response via stimulating T cell, altered tumor microenvironment, and displayed antimetastatic properties. For evaluating its antitumoral and antimetastatic properties, turmeric extract was applied for the first time *in vivo* and *in vitro*. These results suggested that turmeric extract can be used as an essential factor for the treatment or prevention of metastasis in colorectal cancer patients (Li et al., 2018b).

As mentioned earlier, the poor water solubility of curcumin has limited its bioavailability after oral intake and therefore, correlated with low efficacy of therapeutic usage of this compound (Wang et al., 1997; Aggarwal et al., 2007). For overcoming pharmacokinetic and bioavailability restrictions of curcumin's oral administration, investigators have formulated a curcumin liposome for intravenous application and introduced an optimal drug delivery strategy (Li et al., 2005; Li et al., 2007; Mach et al., 2009; Helson et al., 2012; Ranjan et al., 2013; Storka et al., 2015). Different studies evaluated the efficacy of liposomal curcumin anticancer activities in pancreatic cell lines (MiaPaCa-2 or BxPC-3) xenograft models (Li et al., 2005; Mach et al., 2009; Ranjan et al., 2013). Two and eighth after liposomal curcumin constant infusions in beagle dogs, Helson et al. evaluated curcumin and its active metabolite tetrahydrocurcumin pharmacokinetics, organ, and tissue distribution. Results demonstrated that 2h infusions caused greater curcumin and tetrahydrocurcumin plasma concentrations compared with 8h infusions (Helson et al., 2012), although after the 8h infusions, organ and tissue curcumin and THC distributions were overall higher than 2 h infusions (Matabudul et al., 2012). Curcumin liposomes were tested in a placebo-controlled, randomized, dose-escalation, double-blind study in 49 healthy female and male subjects. Volunteers received an individual IV dose of curcumin liposomes (10–400 mg/m^2^; *n* = 2–6 per group, total *n* = 39) or placebo (*n* = 10) for 2 h. It was tolerated well, but at dosages ≥120 mg/m^2^, a transient red blood cell echinocyte formation occurred with both liposomes and curcumin. Besides, the mean cellular volume was increased (Storka et al., 2015).

In a study, tolerability and safety of increasing concentrations of liposomal curcumin were investigated in metastatic cancer patients (Greil et al., 2018). Curcumin pharmacokinetics and anticancer activities were investigated as secondary aims in different studies. This clinical trial was conducted as a phase I, open-label study, and single-center. The liposomal curcumin intravenous infusion was performed weekly in patients with metastatic tumors for 8 weeks. Initiate dose was 100 mg/m^2^ over 8 h, following continuous increasing concentrations, the dose raised to 300 mg/m^2^ over 6 h. At 100–300 mg/m^2^ concentrations over 8 h, no dose-limiting toxicity was reported in 26 subjects. While hemolysis was developed in one of the patients who received 300 mg/m^2^ over 6 h, furthermore in three patients who received the same concentrations hemoglobin was decreased> 2 g/dl. Pharmacokinetic analyses demonstrated that after the infusion, plasma concentrations of curcumin were decreased to undetectable levels. Based on RECIST V1.1, curcumin did not show antitumor activities. Transient clinical benefit and tumor marker responses improved significantly in two subjects. The maximum tolerated concentration of liposomal curcumin was 300 mg/m^2^ over 6 h in pretreated patients, and therefore, starting with this dose is suggested for performing anticancer clinical trials (Greil et al., 2018). Table 3 lists various studies on antimetastatic effects in colorectal cancer.

**TABLE 3:** Curcumin-induced antimetastatic effects against colorectal cancer.

Type of curcumin	Dose	Target	Model	Cell line	Sample size	Results	Ref.
Curcumin	2.5–75 µM	Metastatic features	*In vitro*	HCT-116 and LoVo	-	- Increased metastasis of cancer cells	Calibasi-Kocal et al. (2019)
Curcumin	3, 6, and 9 μg/ml in vitro and 200 mg/kg in vivo	CDC42, RhoA, Rac123,TIMP2, FAK, β-catenin, p-STAT3, STAT3, JNK, *p*-JNK, Erk, *p*-Erk, Src, *p*-Src, Akt, *p*-Akt, Rock, N-Cadherin, and E-Cadherin	*In vitro*, In vivo	HCT116, HT-29, and colon 26-M01	3	- Exhibited cytotoxic effect, inhibited colony formation, decreased cell motility, migration, and epithelial–mesenchymal transitions	Li et al. (2018b)
Liposomal curcumin	100 mg/m^2^ over 8 h, then increased to 300 mg/m^2^ over 6 h	NA	Human	-	32	- 300 mg/m^2^ liposomal curcumin over 6 h was the maximum tolerated dose in these heavily pretreated patients	Greil et al. (2018)
Curcumin	1, 5, 10, 15, 20, 25, 30, and 50 μM	MYC, insulin, and IGF-1 receptors	*In vitro*	SW480	-	- Decreased in the proliferation and migration through downregulation of the insulin and insulin-like growth factor-1 receptors	Hosseini et al. (2019)
Calebin A	0.01, 0.1, 1, 2, 5, and 10 µM	TNF-β and p65-NF-κB	*In vitro*	HCT116, RKO, and SW480	-	- Suppressed NF-B mediated proliferation, invasion, and metastasis	Buhrmann et al. (2019)
Curcumin	0, 5, 10, and 20 μM	FAK, Sp-1, ADEM10, calmodulin, EPHB2, HDAC4, SEPP1, CD24, and E-cadherin	*In vitro*, In vivo	HCT-116, HT-29, HCT-15, HCC-2998, Colo205, Km-12, and SW-620	-	- Suppressed metastasis via Sp-1, FAK inhibition, and E-Cadherin upregulation	Chen et al. (2013)
Curcumin	5–30 μM	miR-21	*In vitro*	Rko and HCT116	-	- Inhibited invasion and metastasis through regulation miR-21 expression	Mudduluru et al. (2011)
F36 and curcumin	10 μM curcumin 1, 3, and 10 μM F36	CDX2, caspase-3, PARP, cleaved PARP, *p*-eIF2a, CHOP, cyclin D1, SERCA2, ATF4, *p*-JNK, *p*-ERK, p-p38, *p*-AKT, SI, and β-actin	*In vitro*, In vivo	SW480, SW620, HCT116, Caco2, HT-29, and HT-29 gal	8	- F36 exhibited more potent inhibitory effect in colorectal cancer cells than curcumin through inhibiting SERCA2 expression	Fan et al. (2014)
Curcumin	5 µM	β1-integrin, ICAM-1, TGF-β3, *p*-Smad2, cyclin D1, Ki-67, vimentin, NF-κB, MMP-13, CD133, CD44, ALDH1, E-cadherin, β-actin, MMP-1, and MMP-9	*In vitro*	HCT116and MRC-5	-	- Modulation of crosstalk between colon cancer stem cells and stromal fibroblasts by curcumin could be a potential therapy for CRC and suppress metastasis	Buhrmann et al. (2014)
Curcumin	5, 10, 20, and 40 μmol/L	NKD2, CXCR4, E-cadherin, Wnt signaling, β-catenin, β-actin, axin, and TCF4	*In vitro*	SW620	-	- Inhibits tumor epithelial–mesenchymal transition by downregulating the Wnt signaling pathway and upregulating NKD2 expression	Zhang et al. (2016)
Curcumin	0.1, 1, 5, 10, and 20 μM	CXCR4, MMP-9, NF-κB	*In vitro*	HCT116, HCT116R	-	- Curcumin potentiates and chemosensitizes HCT116R cells to 5-FU-based chemotherapy	Shakibaei et al. (2015)
Curcumin	10, 20, and 50 μM	Fatty acid synthase and histone H4	*In vitro*	SW480 and SW620	-	- Antimetastatic effect	Lee et al. (2015b)
Difluorinated-curcumin (CDF)	100 nM	miR-21, PTEN, Akt, *p*-Akt, and β-actin	*In vitro*	HCT116, HT-29, and SW620	-	- Inhibit the growth of metastatic colon cancer cells through normalization of miR-21-PTEN-Akt pathway	Roy (2013)
Curcumin	0–50 µM	NF-κB, uPA, and MMP-9	*In vitro*	SW480 and LoVo	-	- Suppressed cancer cell invasion via AMPK-induced inhibition of NF-κB, uPA activator, and MMP9	Tong et al. (2016)
Curcumin	5, 10, 20, and 40 μM	MMP-9 and E-cadherin	*In vitro*	HCT-116	-	- Synergism from the combination of ulinastatin and curcumin showed greater inhibition against colorectal cancer liver metastases through modulating matrix metalloproteinase-9 and E-cadherin expression	Shen et al. (2014)
Curcumin	10 and 50 μM *in vitro* and 1 g/kg *in vivo*	NF-kB, cyclin D1, c-myc, bcl-2, Bcl-xL, cIAP-1, COX-2, ICAM-1, MMP-9, CXCR4, and VEGF	*In vitro*, In vivo	HCT 116	8	- Sensitized cancer cells to capecitabine by modulation of cyclin D1, COX-2, MMP-9, VEGF, and CXCR4 expression	Kunnumakkara et al. (2009)
Curcumin-loaded polymeric micelles, thermosensitive hydrogel system, and its free form	50 mg/kg *in vivo*/20 and 200 μg/ml *in vitro*	NA	*In vitro*, In vivo	CT26	10	- Inhibited tumor growth and metastasis, and prolonged survival of tumor-bearing mice	Zhang et al. (2015)
Curcumin	10, 20, and 50 μM	Fatty acid synthase and histone H4	*In vitro*	SW480 and SW620	-	- Antimetastatic components revealed by the current proteomic analysis	Lee et al. (2014)
Dendrosomal curcumin	0–30 µM	Hef1-1, Zeb1, Claudin 1, and Gapdh	*In vitro*	SW480	-	- Inhibited metastatic potential of cancer cells through downregulation of Claudin1, Zeb1, and Hef1-1 gene expression	Esmatabadi et al. (2015)
Curcumin	5 or 25 μM	IL-8, NF-κB, ERK, and AP-1	*In vitro*	HCT116, HT29	-	- Inhibited neurotensin-mediated interleukin-8 production and migration	Wang et al. (2006)
FLLL32 and curcumin	10, 25, and 50 μM	Bcl-xL, caspase-3, survivin, and STAT3	*In vitro*	DLD-1, HCT-116, and SW480	-	- Inhibited cell viability and induced apoptosis through suppression of STAT3 phosphorylation	Lin et al. (2011)
Curcumin nanofibrous microspheres	2 μg/ml	NA	*In vitro*, In vivo	CT26 and L929	6	- Increased induction of apoptosis in tumor cells and inhibition of tumor angiogenesis for treating abdominal metastases	Fan et al. (2016)
CDF	100 nM	miR-34a,b,c	*In vitro*	SW620, HCT116CR, HCT116p53^−/−^, and HCT116wt	-	- Re-expression of miR-34a and miR-34c, which was consistent with inhibition of cell growth	Roy et al. (2012)
Curcumin	0, 1, 3, and 10 µM	Bcl-2 and miR-497	*In vitro*	HCT8 and HCT8/DDP	-	- Restrained proliferation and facilitated apoptosis	Zheng et al. (2020)
Curcumin	25 µM	hsp27, hsp70, β-actin, cytochrome *c*, Smac, AIF, caspase-3, -8, -9, PARP, and DFF45	*In vitro*	SW480	-	- Induced apoptosis via activation of caspases 3 and 9 which was inhibited by hsp70	Rashmi et al. (2004)
Palladium complexes with 1,7-bis(2-methoxyphenyl)hepta-1,6-diene-3,5-dione	NA	CD133, DLD-1	*In vitro*	HT-29	-	- Exhibited antitumor effect and the hepatic metastasis of a colorectal carcinoma	Fischer-Fodor et al. (2017)

### Regulation of Metastasis in Oral Cancer by Curcumin

Slug, Twist, and Snail transcription factors have been introduced as direct suppressors of E-cadherin through interacting between their COOH-terminal with a 5′-CACCTG-3′ sequence in the promoter of E-cadherin (Yang et al., 2004). Various studies have reported that numerous EMT modulators, including Twist, Slug, E-cadherin, and Snail, have pivotal functions in inhibiting cancer invasion and metastatic capabilities. Through decreasing the level of E-cadherin expression, overexpression of Snail or Twist has been reported to be an essential agent for induction of tumor progression, further aberrant regulation of Snail or Twist has also been demonstrated in different types of epithelial tumors, such as gastric (Rosivatz et al., 2002), prostate (Yuen et al., 2007), breast (Martin et al., 2005), head and neck cancer (Yang et al., 2008). Emerging evidence has shown that E-cadherin downregulation is reversely connected with general survival in patients with different epithelial tumors (Richmond et al., 1997; Zhou et al., 2002; Faleiro-Rodrigues et al., 2004; Tseng et al., 2010; Huber et al., 2011). For example, Fan et al. reported expression levels of Snail and Twist were upregulated. In contrast, E-cadherin expression level was downregulated in oral squamous cell carcinoma (OSCC) patients. Besides, E-cadherin lower expression has been introduced to be an individual prognostic marker in patients with OSCC (Fan et al., 2013).

Lee et al. evaluated curcumin antiinvasive properties on the level of MMPs expression and EMT modulators in OSCC SCC-25 cell line (Lee et al., 2015a). Their results demonstrated that MMP-2 and MMP-9 expression significantly decreased, and hence, invasiveness of oral cancer cells was inhibited after curcumin treatment. Curcumin strongly regulated the expression level of EMT-related factors, including Twist, Snail, and E-cadherin. Besides, curcumin treatment upregulated the p53 expression level, which has been demonstrated to be a crucial factor for repressing EMT. Throughout, it can be said that curcumin has critical functions for preventing cancer development, invasion, and metastasis in oral cancer (Lee et al., 2015a).

Epidermal growth factor receptor (EGFR) is a transmembrane protein with 170-kDa molecular weight, and ErbB family member belongs to receptor tyrosine kinases. It has an extracellular ligand-binding, a transmembrane, and an intracellular domain, which have been reported to be associated with tyrosine kinase activity. Binding of ErbB to epidermal growth factor (EGF) as its primary ligand resulted in the EGFR-mediated formation of heterodimers or homodimers with other ErbB protein family members, including ErbB2, ErbB3, and ErbB4. This causes autophosphorylation, which led to the downstream signaling activation, including MAPK/Ras/Raf/ERK, JAK2/STAT3, and PI3K/Akt/mTOR pathways. These signaling pathways have crucial roles in regulating cancer cell proliferation, differentiation, invasion, and migration (Yarden and Sliwkowski, 2001). Studies have shown that activity and upregulation of EGFR are strongly related to increased tumor invasiveness, multiplication, and metastasis (Doumiati et al., 2012; Lin et al., 2014a). Poor prognosis and inadequate responses to chemotherapy have been observed in positive pEGFR tumors (Aquino et al., 2012). Hence, EGFR has been introduced as an essential anticancer drug target (Rabinowits and Haddad, 2012). Classic EGFR inhibitors have been divided into two major categories, including anti-EGFRs monoclonal antibodies and cetuximab, panitumumab, and trastuzumab are anti-EGFRs monoclonal antibodies, while gefitinib, erlotinib, and lapatinib are categorized as tyrosine kinases inhibitors. Currently, the mentioned drugs are used for the treatment of some epithelium-originated carcinomas, such as colorectal (Soeda et al., 2014), head and neck (Maseki et al., 2013), lung (Sgambato et al., 2014), and breast cancer (Nechushtan et al., 2014). Moreover, because of their extended side effects and chemoresistance, the application of these inhibitors has been very limited in treating most patients with cancer.

Through decreasing Egr-1 *trans*-activation activities, it has been shown that curcumin inhibited the EGFR-mediated growth of human colon cancer cells (Chen et al., 2006). Furthermore, via repressing EGFR expression, cell apoptosis was significantly promoted in breast cancer cells after curcumin treatment (Sun et al., 2012).

Zhen et al. studied the effectiveness of curcumin on invasion and proliferation in SCC-25 cell lines (Zhen et al., 2014). Based on their results, considerable inhibitory effects were observed from curcumin on the SCC-25 cells’ proliferation. Furthermore, curcumin caused the arrest of the cell cycle in the G2/M phase in a dose-dependent manner. Curcumin downregulated the MMP-2, MMP-9, uPA, and uPAR expression, which led to the inhibited invasion of SCC-25 cells. Curcumin downregulated the MMP-9, MMP-2, uPAR, and uPA expression, which led to the inhibited invasion of SCC-25 cells. It has also been revealed that curcumin regulated Akt, ERK1/2, and STAT3 as downstream signaling targets of *p*-EGFR and EGFR. Besides, as the most important finding of their study, EGF-triggered EGFR phosphorylation and EGF-mediated invasion of SCC-25 cells were suppressed after treatment with curcumin (Zhen et al., 2014).

In various cancer types, hepatocyte growth factor (HGF) and c-Met, its receptor, have been explained to be implicated in the EMT program (Huang et al., 2019). Remarkably, emerging evidence has shown that the HGF/c-Met signaling pathway activation promoted cancer cell migration and invasiveness. Chemopreventive and antitumoral activities of Curcumin have been comprehensively investigated in colorectal cancer (Ismail and Othman, 2019; Wong et al., 2019). Moreover, curcumin has been introduced to be a prominent EMT suppressor in various cancers (Jiao et al., 2016; Liang et al., 2017; Wang et al., 2017; Bahrami et al., 2019b).

In a study, Ohnishi et al. studied the potential impacts of curcumin on HGF-induced EMT in OSCC (Ohnishi et al., 2020). They reported that via activating HGF receptor c-Met, and downstream ERK pathway in HSC4 and Ca9-22 OSCC cell lines, the HGF signaling pathway induced EMT process. Furthermore, via repressing c-Met, HGF-mediated EMT and cell motility were inhibited by curcumin treatment in HSC-4 and Ca9-22 cells. Through downregulating the expression levels of phosphorylated c-Met, and ERK, curcumin effectively inhibited the HGF-triggered upregulation of vimentin. Taking everything into account, the findings of the reviewed investigation demonstrated that possibly by inhibiting c-Met expression, curcumin has pivotal roles in reversed regulating of HGF-induced EMT in oral cancer cells (Ohnishi et al., 2020). Table 4 lists various studies on antimetastatic effects in oral cancer.

**TABLE 4:** Efficacy of curcumin for triggering metastasis in oral cancer.

Type of curcumin	Dose	Target	Model	Cell line	Sample size	Results	Ref.
Curcumin	0–15 µM	p53, Snail, Twist, E-cadherin, MMP-2, MMP-9	*In vitro*	SCC25	-	- Inhibited invasiveness and epithelial–mesenchymal transition through reducing MMP 2, 9 and modulating p53-E-cadherin pathway	Lee et al. (2015a)
Curcumin	0, 10, 20, 40, 80 μmol/L	EGFR, P-EGFR, Akt, ERK1/2, STAT3, MMP-2, MMP-9, uPA, and uPAR	*In vitro*	SCC-25	-	- Inhibited cell proliferation and invasion via EGFR signaling pathways	Zhen et al. (2014)
Curcumin	0, 5, 10, 20, 30, 60, and 100 μmol/L	cdc27, EGFR substrate 15, and PPAR-α	*In vitro*	SCC-4	-	- Antiproliferative and antimetastatic effects	Chen et al. (2011)
Curcumin	2, 5, 10, 20, 30, 40, and 50 µM *in vitro* and 70 mg/kg *in vivo*	NA	*In vitro*, In vivo	HACAT, CAL27, SCC25, and NIH-3T3	NA	- Inhibited cell migration	de Campos et al. (2017)
Curcumin	15 µM	ERK, c-met, E-cadherin, vimentin, pro-MMP2, pro-MMP9	*In vitro*	HSC4 and Ca9-22	-	- Inhibits epithelial–mesenchymal transition in cancer cells via c-Met blockade	Ohnishi et al. (2020)
Curcumin	20 µM	Snail, β-actin	*In vitro*	OECM-1, HOKs	-	- Reduced tumors differentiation and metastasis through downregulation of Snail expression	Lee et al. (2013)
Curcumin	0–100 mmol/L	MMP-2, MMP-9	*In vitro*	Tca8113	-	- Suppressed invasion and migration by reducing the activities of MMP-2 and MMP-9	Wang et al. (2008)

### Regulation of Metastasis in Esophageal Cancer by Curcumin

As an important member of the CXC subfamily, stromal cell-derived factor 1a (SDF-1a) interacts with the C-X-C chemokine receptor type 4 (CXCR4), seven-transmembrane G protein-coupled receptor (7TMGPCR). It is also a principal agent in the induction of metastasis and invasion in cancer cells (Vandercappellen et al., 2008). Various *in vivo* studies have shown that SDF-1a is a key agent for triggering migration and metastasis in breast cancer cells (Müller et al., 2001). Moreover, suppression of CXCR4 gene expression mediated by Short hairpin RNA (shRNA) significantly inhibited cell proliferation of esophageal carcinoma (EC). Upregulation of CXCR4 has been reported to be related to poor prognosis, poor clinical outcomes, and raised metastatic features in EC cells (Kaifi et al., 2005).

Lipid rafts are plasma membrane firmly packed microdomains that consist of dynamic assemblies of cholesterol, glycosphingolipid, and proteins. The rafts can affect signal transduction via the recruitment of membrane-associated protein kinases (Zajchowski and Robbins, 2002). The interaction between the growth factors and their receptors plays a critical role in specific adapter proteins and protein kinases translocation to lipid rafts. It provides essential signals for the regulation of cell proliferation (Pike, 2005). Numerous authors demonstrate that the CXCR4 connection with lipid rafts is involved in effective signaling processes (Chinni et al., 2008; Altenburg and Siddiqui, 2009). Ectopic expression of a CXCR4-GFP fusion protein in CXCR4-deficient human hematopoietic progenitor cell lines showed that lipid rafts are crucial in the SDF-1a-induced CXCR4 signaling and surface expression (van Buul et al., 2003).

The colocalization of Rho GTP-binding protein Rac1 and CXCR4 in lipid rafts resulted in increased activation of Rac1 GTPase. It also led to the sensitivity of hematopoietic cells to SDF-1α (Wysoczynski et al., 2005). SDF-1a/CXCR4 axis-mediated cell migration needs the activation of phosphatidylinositol 3- kinase (PI3K)/protein kinase B (Akt) in breast cancer cells (Lee et al., 2004). Numerous investigations demonstrated that hormones, growth factors, and cytokines stimulated the MMP-2 expression via nuclear factor-kappaB (NF-kB) activation (Han et al., 2001; Philip et al., 2004). NF-kB transcription factor constitutive activation in EC has been related to metastatic ability and chemotherapy resistance (Izzo et al., 2006). Besides, active Rac1 (GTP bound Rac1; GTP-Rac1) is required for collagen-mediated MMP-2 activation (Zhuge and Xu, 2001).

Lin et al. investigated these features by evaluating the CXCR4 suppression effects along with a CXCR4-neutralizing antibody and the CXCR4-specific inhibitor AMD3100 treatments (Lin et al., 2014b). Curcumin prevents SDF-1α-mediated cell migration, cell surface localization of CXCR4 at lipid rafts, the activity of matrix metalloproteinase-2 (MMP-2) promoter, and also signaling of lipid raft-related ras-related C3 botulinum toxin substrate 1 (Rac1)/phosphatidylinositol 3-kinase (PI3K) p85α/Akt. Furthermore, curcumin inhibits SDF-1α-mediated cell metastasis by knockdown of the signaling complex of Rac1-PI3K at lipid rafts but did not abolish lipid raft generation. Researchers also explain that the decreasing lipid raft-related Rac1 activity by curcumin was the requirement for preventing cell surface localization of CXCR4 at lipid rafts, SDF-1α-mediated PI3K/Akt/NF-κB activation, cell invasion, and MMP-2 promoter activity. Overall, their findings displayed that curcumin hinders SDF-1α-mediated EC cell invasion by suppressing the lipid raft formation-related Rac1-PI3K-Akt signaling complex, MP-2 promoter activity, likely through the inhibition of Rac1 activity, and the cell surface localization of CXCR4 with lipid rafts (Lin et al., 2014b).

## Conclusion

For centuries, curcumin has been widely used as a dietary spice, and contemporary research studies have confirmed its efficacy in cancer therapy. Anticancer effects are the most pivotal properties of curcumin, which affect the different stages of cancer progression, including cancer cell formation, proliferation, and tumor invasion. According to extensive research, curcumin can suppress metastasis in GI cancers via regulating various signaling pathways. Curcumin has a crucial function in metastasis prevention by several mechanisms, including preventing transcription factors as well as their signaling pathways (e.g., NF-κB, STAT3, AP-1), multiple proteases (e.g., MMPs, uPA), inflammatory cytokines (e.g., CXCL1, IL-6, CXCL2, IL-8), modulation of miRNAs (e.g., miR181b, miR21), multiple protein kinases (e.g., FAK, MAPKs), and heat shock proteins (HLJ1). According to research, curcumin treatment leads to significant elevation in metastatic tumor cross-sectional volume (70%) and zone (46%). Curcumin may enhance LLC's metastatic growth in mice by elevating the concentration of VEGF, angiogenic factors, monocyte chemotactic protein-1 (MCP-1), and IL-1β.

In addition to the possible pharmaceutical effects of curcumin, the safety and relevant therapeutic dose should also be established, given reports on the side effects of curcumin in cancer therapy. Future clinical and preclinical investigations on anti-metastasis properties of curcumin should be designed in a way that indicates the safety and effectiveness of curcumin in inhibiting cancer metastasis.

It has been found that curcumin nanoparticles improve their anticancer properties via cancer cellular uptake enhancement, targeted zone internalization, and high bioavailability. Polymeric nanoparticles, polymeric micelles, and liposomes are the regular nano-carriers exploited for curcumin encapsulation. More efforts are needed to improve curcumin nanoparticles for targeted therapy of cancer cells (e.g., nanoparticle coating with peptides/antibodies that bind to upregulated receptors on the cancer cells’ surface) in the future.
